# Illuminating the future: Enhanced glowing plants achieved by rewiring metabolism

**DOI:** 10.1093/plcell/koae286

**Published:** 2024-10-22

**Authors:** Andrew C Willoughby

**Affiliations:** Assistant Features Editor, The Plant Cell, American Society of Plant Biologists; Department of Biology, Duke University, Durham, NC 27708, USA

A glowing plant, bright enough to read by, may not be limited to science fiction due to new research into autoluminescent plants by **Jieyu Ge, Xuye Lang, Jiayi Ji, Chengyi Qu, He Qiao, and colleagues (Ge et al. 2024)**. Although the first glowing plant was developed nearly 40 years ago (Ow et al. 1986), it required supplementation with the substrate luciferin, which can be cleaved by the enzyme luciferase (Luz) to produce light and therefore was not autoluminescent. Recently, a fungal bioluminescence pathway (FBP) was identified that allows for autoluminescent plants. The FBP requires only 2 enzymes in a multistep pathway to produce the Luz substrate hispidin from the plant metabolite caffeic acid. The FBP also has the advantage of a recycling step that regenerates caffeic acid (Kotlobay et al. 2018). Caffeic acid is an intermediate in the biosynthesis of the structural cell wall polymer lignin, and its availability limits the intensity of these autoluminescent plants (Mitiouchkina et al. 2020).

To tackle this issue, Ge et al. standardized and investigated published transcriptomics data from poplar, a model system for lignin production. They determined key genes involved in caffeic acid and hispidin metabolism to guide multiplex gene-silencing strategies and identified the most effective candidates in transient expression experiments. By combining artificial microRNA expression and the additional introduction of a tyrosine-derived caffeic acid biosynthesis pathway, the authors achieved increased caffeic acid levels and brighter autoluminescent plants (see Fig.).

**Figure.**
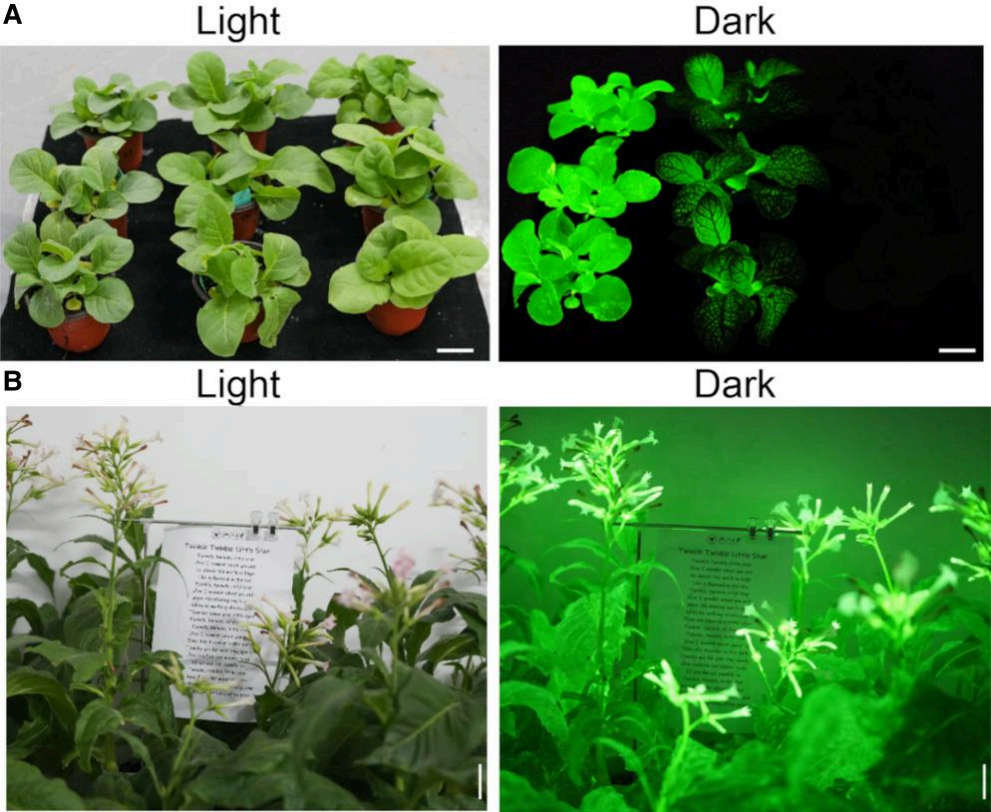
Enhanced autoluminescent plants. **A)** eFBP2 plants (left row) were compared with previously published eFBP plants (middle row; Zheng et al. 2023) and wild-type plants (right row). **B)** Performance of stably transformed autoluminescent eFBP2 tobacco plants with a 10-s camera exposure. Figure adapted from Ge et al. (2024), Figure 4.

The authors found that the efficiency of light production in the enhanced FBP (eFBP2) plants is still quite low compared with the energy gained from photosynthesis. This means there is plenty of room for improvement in future iterations, paving the way for even brighter and more efficient glowing plants, which can be used for novel research applications (Calvache et al. 2024). This approach of using computational modeling and ever-increasing transcriptomics data may also enable the engineering of plant metabolism for natural product production, nutrition, and plant defense.

## Dive Curated Terms

The following phenotypic, genotypic, and functional terms are of significance to the work described in this paper:

caffeic acid CHEBI: CHEBI:36281

## References

[koae286-B1] Calvache C , Vazquez-Vilar M, Moreno-Giménez E, Orzaez D. A quantitative autonomous bioluminescence reporter system with a wide dynamic range for Plant Synthetic Biology. Plant Biotechnol J. 2024:22(1):37–47. 10.1111/pbi.14146 37882352 PMC10754000

[koae286-B2] Ge J , Lang X, Ji J, Qu C, Qiao H, Zhong J, Luo D, Hu J, Chen H, Wang S, et al Integration of biological and information technologies to enhance plant autoluminescence. Plant Cell. 2024. First published 21 August 2024. 10.1093/plcell/koae236 PMC11530770 39167833

[koae286-B3] Kotlobay AA , Sarkisyan KS, Mokrushina YA, Marcet-Houben M, Serebrovskaya EO, Markina NM, Gonzalez Somermeyer L, Gorokhovatsky AY, Vvedensky A, Purtov KV, et al Genetically encodable bioluminescent system from fungi. Proc Natl Acad Sci U S A. 2018:115(50):12728–12732. 10.1073/pnas.1803615115 30478037 PMC6294908

[koae286-B4] Mitiouchkina T , Mishin AS, Somermeyer LG, Markina NM, Chepurnyh TV, Guglya EB, Karataeva TA, Palkina KA, Shakhova ES, Fakhranurova LI, et al Plants with genetically encoded autoluminescence. Nat Biotechnol. 2020:38(8):944–946. 10.1038/s41587-020-0500-9 32341562 PMC7610436

[koae286-B5] Ow DW , Wood KV, DeLuca M, de Wet JR, Helinski DR, Howell SH. Transient and stable expression of the firefly luciferase gene in plant cells and transgenic plants. Science. 1986:234(4778):856–859. 10.1126/science.234.4778.856 17758108

[koae286-B6] Zheng P , Ge J, Ji J, Zhong J, Chen H, Luo D, Li W, Bi B, Ma Y, Tong W, et al Metabolic engineering and mechanical investigation of enhanced plant autoluminescence. Plant Biotechnol J. 2023:21(8):1671–1681. 10.1111/pbi.14068 37155328 PMC10363767

